# Supplementary material for: Reciprocal antagonism of PIN1-APC/CCDH1 governs mitotic protein stability and cell cycle entry
Source: Nat Commun. 2024 Apr 15;15:3220. doi: 10.1038/s41467-024-47427-w (PMC11018817; doi:10.1038/s41467-024-47427-w)
Supplement: Supplementary file 1 — Supplementary Information [file 41467_2024_47427_MOESM1_ESM.pdf]

Supplementary Information

Reciprocal antagonism of PIN1-APC/C^{CDH1} governs mitotic protein stability and cell cycle entry

Shizhong Ke[1,†], Fabin Dang[2,†], Lin Wang[1,†], Jia-Yun Chen[3,4,†], Mandar T. Naik[5], Wenxue Li[6], Abhishek Thavamani[1], Nami Kim[1], Nandita M. Naik[5], Huaxiu Sui[7], Wei Tang[8], Chenxi Qiu[1,9], Kazuhiro Koikawa[1], Felipe Batalini[1,10], Emily Stern Gatof[1], Daniela Arango Isaza[1], Jaymin M. Patel[1], Xiaodong Wang[11], John G. Clohessy[12], Yujing J. Heng[2], Galit Lahav[3], Yansheng Liu[6,13], Nathanael S. Gray[14], Xiao Zhen Zhou[15]*, Wenyi Wei[2,*], Gerburg M. Wulf[1,*], and Kun Ping Lu[15,*]

[1]Division of Hematology/Oncology, Department of Medicine and Cancer Research Institute, Beth Israel Deaconess Medical Center, Harvard Medical School, Boston, MA 02215, USA.

[2]Department of Pathology, Beth Israel Deaconess Medical Center and Cancer Research Institute, Harvard Medical School, Boston, MA 02215, USA.

[3]Department of Systems Biology, Harvard Medical School, Boston, MA 02215, USA.

[4]Laboratory of Systems Pharmacology, Harvard Medical School, Boston, MA 02215, USA.

[5]Department of Molecular Biology, Cell Biology & Biochemistry, Brown University, Providence, RI 02912, USA

[6]Yale Cancer Biology Institute, West Haven, CT 06516, USA

[7]Key Laboratory of Functional and Clinical Translational Medicine, Fujian Province University, Xiamen Medical College, Xiamen 361023, China.

[8]Data Science & Artificial Intelligence, R&D, AstraZeneca, Gaithersburg, USA

[9]Department of Genetics, Harvard Medical School, Boston, MA 02115, USA.

[10]Department of Medicine, Division of Medical Oncology, Mayo Clinic, Arizona, USA

[11]Molecular and Integrative Physiological Sciences, Department of Environmental Health, Harvard T.H. Chan School of Public Health, Boston, MA 02215, USA.

[12]Preclinical Murine Pharmacogenetics Facility, Beth Israel Deaconess Medical Center, Harvard Medical School, Boston, MA 02215, USA.

[13]Department of Pharmacology, Yale University School of Medicine, New Haven, CT 06510

[14]Department of Chemical and Systems Biology, Chem-H and Stanford Cancer Institute, Stanford University, Stanford, CA 94305, USA.

[15]Departments of Biochemistry & Oncology, Western University, London, ON N6A 3K7, Canada

Content:

Supplementary Fig. 1-10

Supplementary Table 1. The relationship between PIN1 protein expression and overall survival using the dataset by Tang et al.

Supplementary Table 2. The characteristics of the human BC specimens

Supplementary Table 3. Genotyping primers and probes for K14*cre; Brca1wt/f; p53wt/f* transgenic mice

Supplementary Table 4. The sequences of the primers used for qRT-PCR analyses

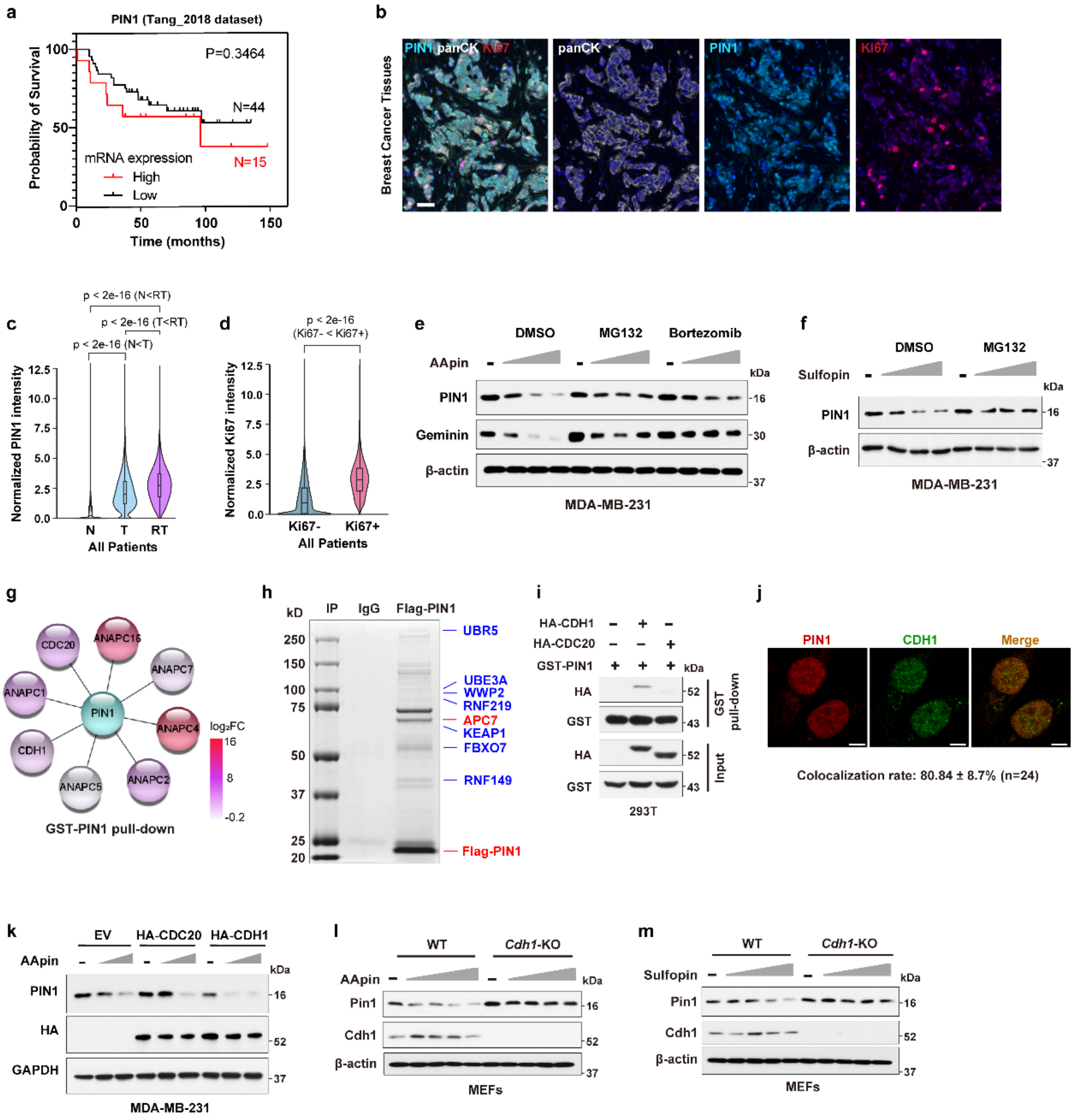

Supplementary Fig. 1 | Cell cycle regulator APC/C^{CDH1} is a physiological E3 ligase for PIN1

a, Overall survival for BC with low and high PIN1 mRNA levels. PIN1-Low, n=44 patients; PIN1-High, n=15 patients. Log rank test, p=0.3464. **b,** Representative images of PIN1 (cyan), panCK (white) and Ki67 (red) in human BC tissue specimens detected by multiplex IHC. Scale bars, 40 µm. **c, d,** PIN1 intensity (**c**) or Ki67 intensity (**d**) at the single-cell level in the whole BC tissue specimens from Fig. 1c were shown in violin plots. Ki67-negative, Ki67 intensity <1. Data were analyzed by one-way ANOVA followed by Tukey's multiple comparisons test (**c**) or unpaired two-sided t-test (**d**). p values are shown. **e, f,** IB analysis for indicated proteins from MDA-MB-231 cells treated with AApin (ATO (0.5, 1, 2 µM) plus ATRA (5, 10, 20 µM)) (**e**) or Sulfopin (2.5, 5, 10 µM) (**f**) for 3 days and 10 µM MG132 or 1 µM Bortezomib for last 12 hrs before harvesting. **g,** Data from PIN1 affinity purification-mass spectrometry were used to generate the interaction networks by Cystoscape. Heatmap

represented log2-transformed fold changes of GST-PIN1 versus GST. **h,** Coomassie blue stain of PIN1-interacting proteins identified by mass spectrometry. MDA-MB-231 cells were transfected with Flag-PIN1 and treated with 10 μM MG132 for 12 hrs. Lysates were immunoprecipitated with M2 (anti-Flag) or IgG beads. **i,** Co-IP of HA-CDH1 with GST-PIN1. 293T cells were transfected with indicated constructs for 36 hrs and GST-PIN1 were pulled down with glutathione beads. **j,** Confocal images of the colocalization of endogenous PIN1 (red) and CDH1 (green). Colocalization rates were calculated by LAS X software. Scale bars, 5 μm. **k,** IB analysis of PIN1 from MDA-MB-231 cells stably expressing HA-CDC20 or HA-CDH1 and treated with AApin (ATO (1, 2 μM) plus ATRA (10, 20 μM)) for 3 days. **l, m,** IB analysis of PIN1 from WT or *CDH1* KO MEFs treated with increasing concentrations of PIN1 inhibitors for 3 days. The images were representative images from 3 independent experiments (**e, f, i, j, k-m**). Source data are provided as a Source Data file.

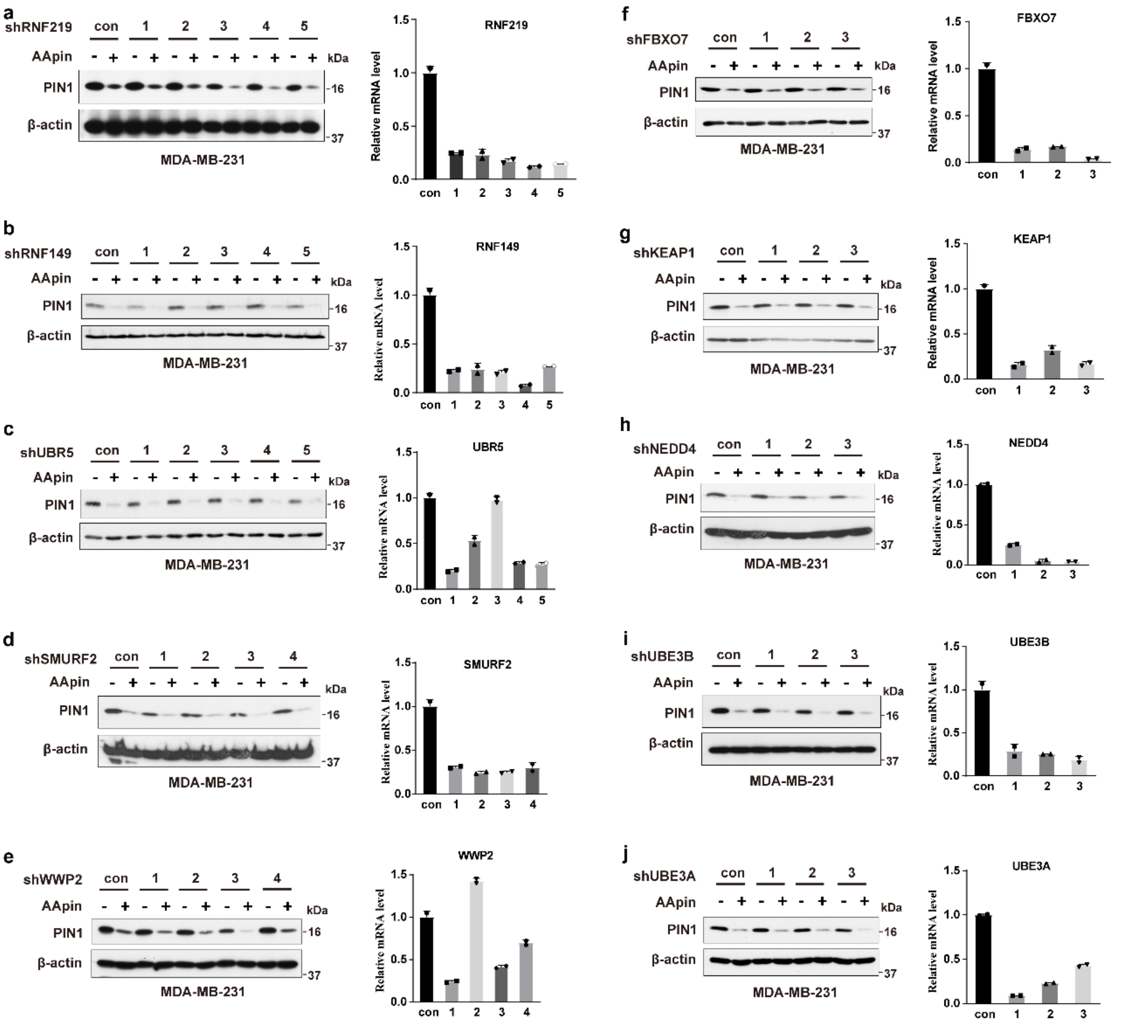

Supplementary Fig. 2 | Cell cycle regulator APC/C^{CDH1} is a physiological E3 ubiquitin ligase for PIN1.

a-j, Validation of potential PIN1-interacting E3 ligases identified by mass spectrometry. IB of PIN1 from MDA-MB-231 cells stably expressing indicated shRNAs and treated with AApin (1.5 μM ATO plus 15 μM ATRA) for 3 days. The knockdown efficiency of the respective shRNA plasmids was validated by RT-PCR. n=2 independent experiments. Data in graphs are mean ± SD. Source data are provided as a Source Data file.

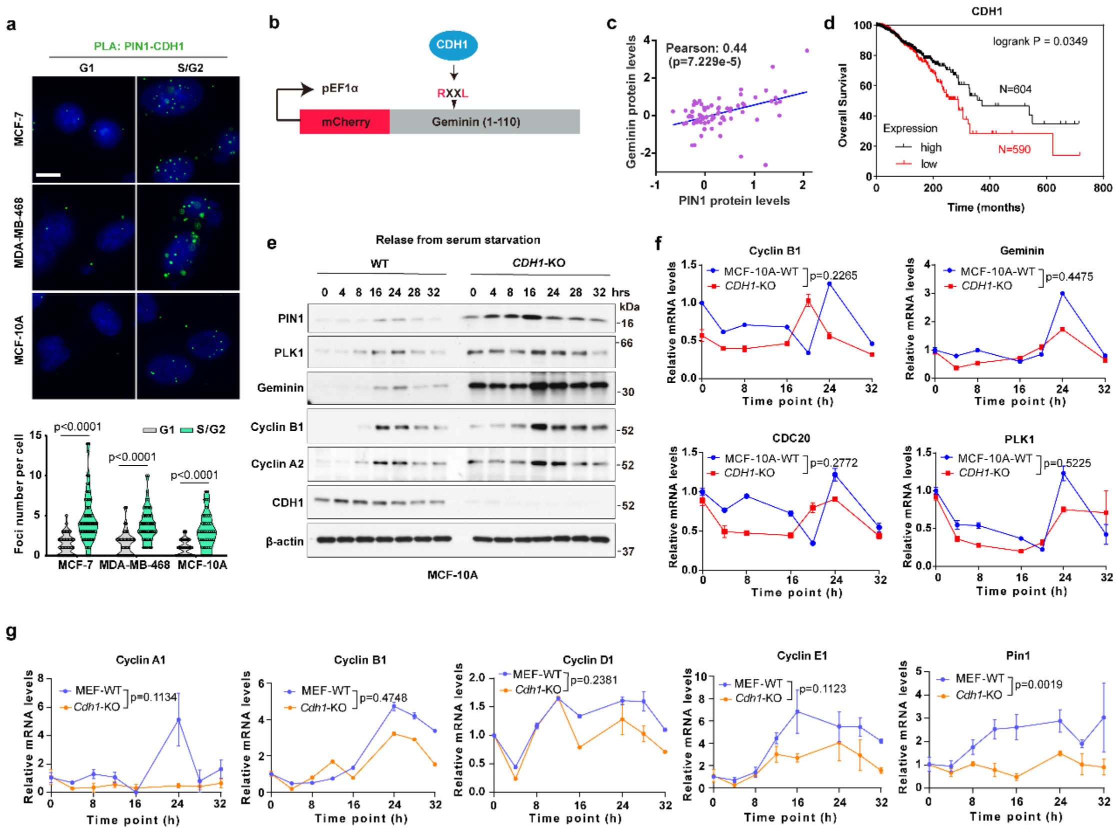

Supplementary Fig. 3 | Cell cycle regulator APC/C^{CDH1} is a physiological E3 ubiquitin ligase for PIN1.

a, Detection of endogenous PIN1-CDH1 interaction by in situ proximity ligation assay (PLA) in the indicated BC cells in different cell cycle stages. Nucleus were visualized by DAPI staining. Scale bars, 5 µm. (bottom panel) Quantification of PLA signals from at least 30 cells were enumerated. Data in graphs are mean ± SD and analyzed by unpaired two-sided t-test. p values are shown. **b,** Schematic diagram of the APC/C-degron reporter (Geminin: aa1–110). **c,** Correlation between PIN1 protein levels and Geminin protein levels across 75 BRCA samples from cBioPortal. Protein level z-scores measured with mass spectrometry by the Clinical Proteomic Tumor Analysis Consortium (CPTAC). **d,** Overall survival for BRCA tumors in TCGA with low and high *CDH1* mRNA levels. CDH1-Low, n=590 patients; CDH1-High, n=604 patients. Log rank test, p=0.0349. **e,** IB analysis of WT and *CDH1* KO MCF-10A cells synchronized in G1 phase followed by releasing back into the cell cycle before harvesting cells at indicated time points. **f,** RT-PCR analysis of indicated mRNA of WT and *CDH1* KO MCF-10A cells synchronized in G1 phase followed by releasing back into the cell cycle before harvesting cells at indicated time points. **g,** RT-PCR analysis of indicated mRNA from WT and *Cdh1* KO MEFs synchronized in G1 phase by serum starvation, followed by releasing back into the cell cycle before harvesting cells at indicated time points. n=3 independent experiments (**f, g**). Data in graphs are mean ±SD and analyzed by unpaired two-sided t-test, p values are shown (**f, g**). The images were representative images from 3 independent experiments (**a, e**). Source data are provided as a Source Data file.

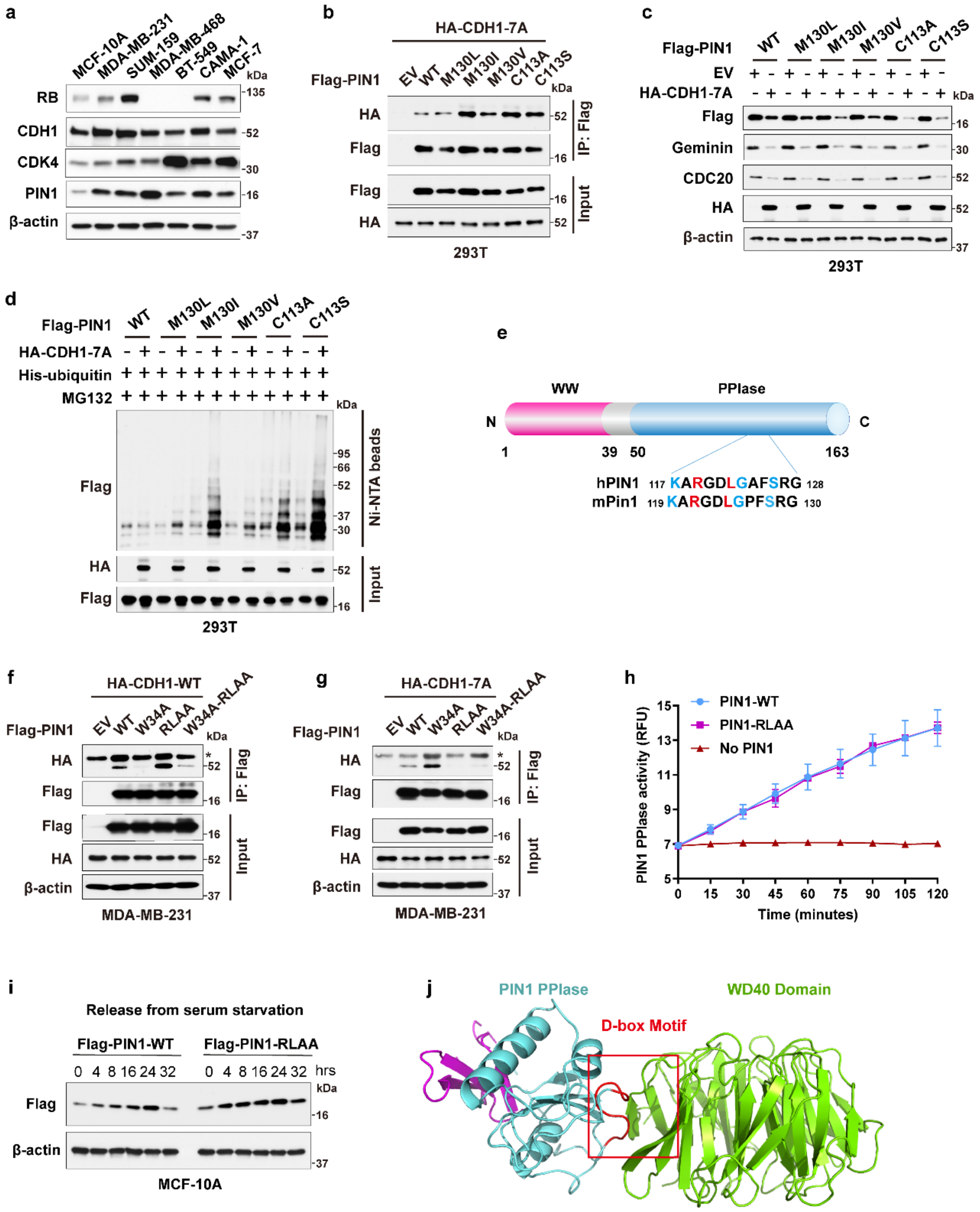

Supplementary Fig. 4 | Constitutively active APC/C^{CDH1} targets PIN1 and other mitotic proteins for degradation to provoke cell cycle exit

a, IB analysis for indicated proteins from multiple BC cell lines. **b,** IB analysis of immunoprecipitates from 293T cells stably co-expressing Flag-PIN1-WT or disabling mutants and CDH1-7A mutant treated with 10 μM MG132 for 12 hrs and pulled down using Flag-M2 beads. **c,** IB analysis for indicated proteins from 293T cells stably co-expressing CDH1-7A or empty vector (EV) in the presence of Flag-PIN1 and its mutants. **d,** IB analysis of the ubiquitinated proteins from 293T cells transfected with the indicated constructs for 48 hours and treated with 2 μM MG132 for 12 hrs and

pulled down by Ni-NTA agarose. **e,** Domain architecture of PIN1 containing an N-terminal WW domain binding specific pSer/Thr-Pro motifs, and a C-terminal peptidyl-prolyl cis/trans isomerase (PPIase) domain that catalyzes prolyl isomerization of specific pSer/Thr-Pro motifs. Note the sequence homology of D-box motifs (RXXL) in human and mouse. **f, g,** IB analysis of immunoprecipitates from MDA-MB-231 cells stably co-expressing indicated constructs and treated with 10 μM MG132 for 12 hrs and immunoprecipitated using Flag-M2 beads. Input is 5% of the total lysates. Non-specific bands for IgG_H marked with asterisks (*). **h,** The enzymatic activities of WT and RLAA mutant PIN1 were determined by fluorescence intensity and data were recorded every 15 min for 120 min. The fluorescence readings were expressed in relative fluorescence units (RFU). n=2 independent experiments. Data in graphs are mean ± SD. **i,** MCF-10A cells were stably expressing Flag-PIN1-WT or Flag-PIN1-RLAA and synchronized in G1 phase followed by releasing back into the cell cycle before harvesting cells at indicated time points. IB analysis for Flag-PIN1. The images were representative images from 3 independent experiments (**a-d, f, g, i**). **j,** A structural modeling illustration of docking the PIN1 PPIase domain (cyan; PDB: 1PIN) to the CDH1-WD40 domain (chartreuse; PDB: 4UI9_R). D-box motif is marked in red. Source data are provided as a Source Data file.

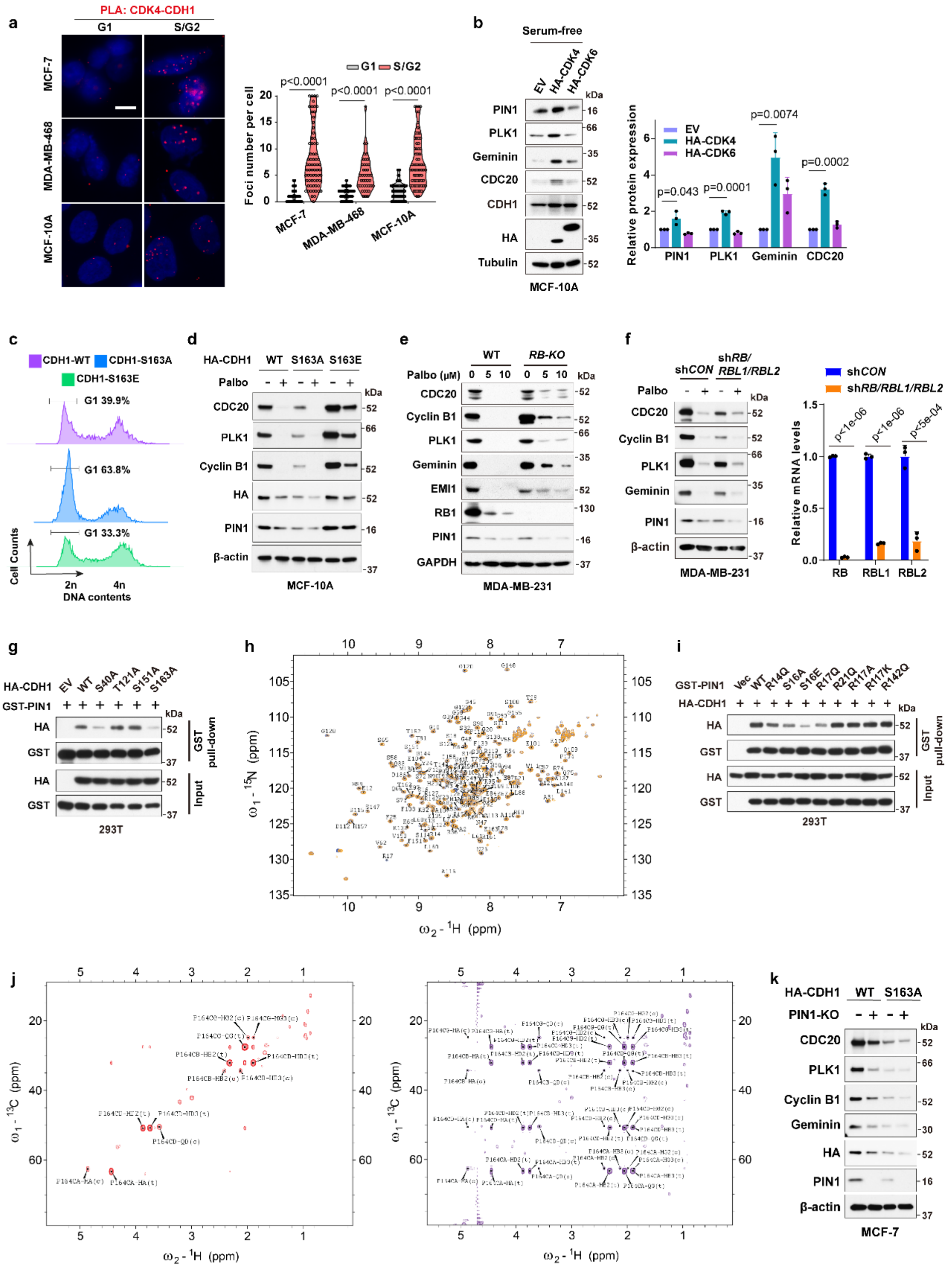

Supplementary Fig. 5 | PIN1 catalyzes *trans* to *cis* isomerization of the pS163-P motif in CDH1 to prevent CDH1 dephosphorylation

a, (Left) Detection of endogenous CDK4-CDH1 interaction by PLA in the BC cells in different cell-cycle stages. Nucleus were stained by DAPI. Scale bars, 5 μm. (Right) Quantification of PLA signals. n>30 cells. Data in graphs are analyzed by unpaired two-sided t-test. **b,** (Left) IB analysis of indicated proteins from MCF-10A transfected with either CDK4 or CDK6 and cultured in serum-free condition for 36 hrs. (Right)

Quantification for the relative levels of the indicated proteins. n=3 independent experiments. Data in graphs are mean ± SD and analyzed by unpaired two-sided t-test. p values are shown (**a, b**). **c,** FACS analysis of DNA contents in MCF-7 cells expressing either CDH1-WT, CDH1-S163A, or CDH1-S163E. **d,** IB analysis of indicated proteins from MCF-10A cells expressing either CDH1-WT, CDH1-S163A or CDH1-S163E and treated with 1 μM palbociclib for 3 days. **e,** IB analysis for indicated proteins from WT or *RB*-KO MDA-MB-231 cells treated with indicated concentration of Palbociclib for 3 days. **f,** IB analysis for indicated proteins from sh*CON* or sh*RB/RBL1/RBL2* MDA-MB-231 cells treated with 5 μM Palbociclib for 3 days. (Right) Knockdown of *RB/RBL1/RBL2* was validated by RT-PCR. n=3 independent experiments. Data in graphs are mean ±SD and analyzed by unpaired two-sided t-test. p values are shown. **g,** IB analysis of GST pull-down precipitates from 293T cells transfected with GST-PIN1 and HA-CDH1 mutants as indicated for 36 hrs. **h,** The overlay of full ^{1}H-^{15}N HSQC spectrum of ^{15}N-labeled PIN1 protein (blue) and its complex with the CDH1 phosphopeptide (orange). **i,** IB analysis of GST pull-down precipitates from 293T cells transfected with HA-CDH1 and GST-PIN1mutants for 36 hrs. **j,** ^{13}C-HSQC (left) and ^{13}C-HSQCOCSY (right) spectrum of ^{13}C-labeled CDH1-P164 for the proline isomer assignments. **k,** IB analysis of indicated proteins from WT or *PIN1* KO MCF-7 cells expressing either WT CDH1 or S163A mutant CDH1. The images were representative images from 3 independent experiments (**a-g, i, k**). Source data are provided as a Source Data file.

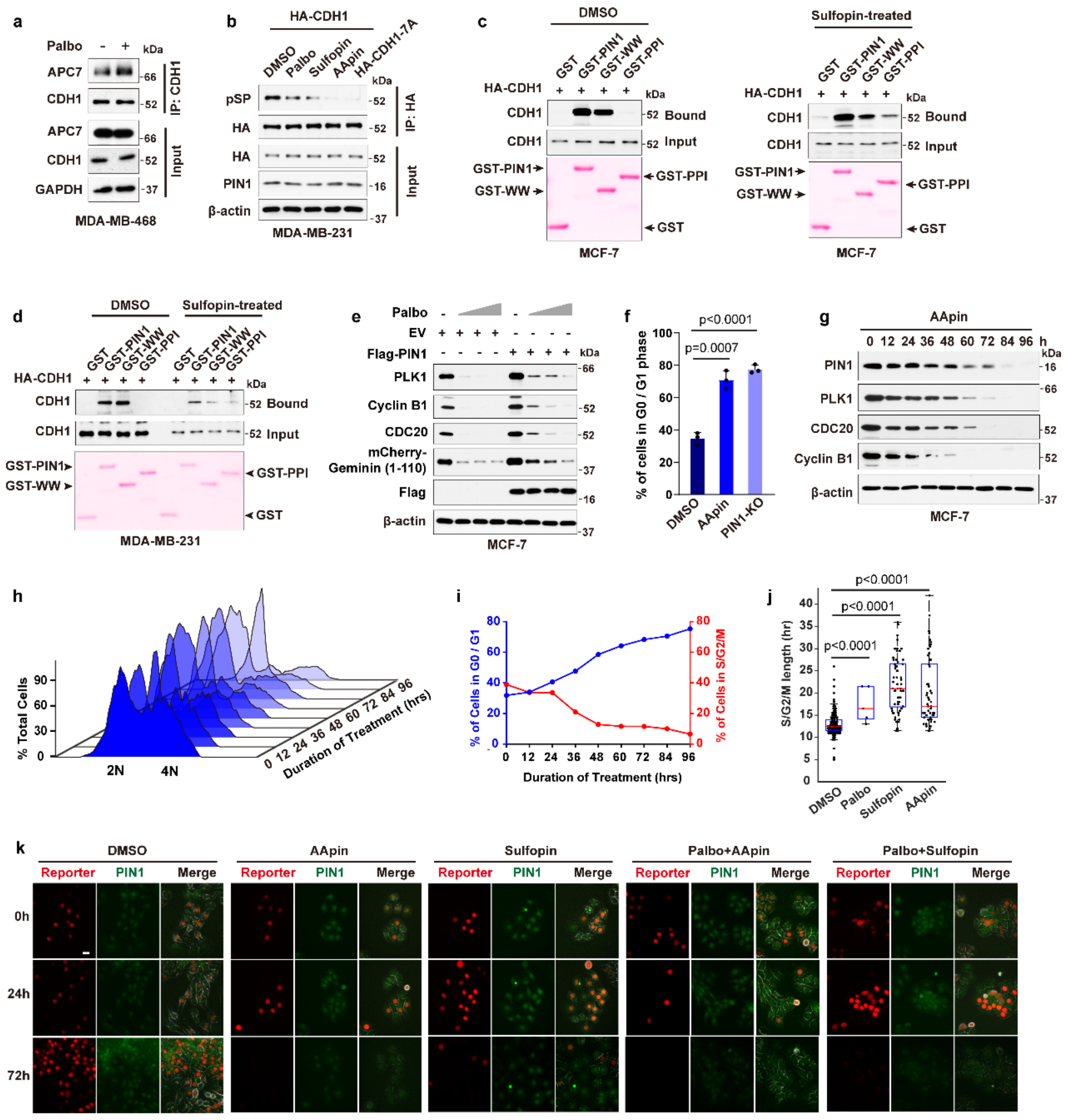

Supplementary Fig. 6 | Pharmacologic inhibition of PIN1 and CDK4 synergistically and irreversibly reactivate APC/C^{CDH1} to induce degradation of PIN1 and other mitotic proteins

a, IB analysis of immunoprecipitates from MDA-MB-468 cells treated with 5 μM Palbociclib and pulled down by anti-CDH1 antibody. **b,** IB analysis of immunoprecipitates from MDA-MB-231 cells stably expressing HA-CDH1 treated with Palbociclib or PIN1 inhibitors and pulled down by anti-HA antibody. Input is 5% of the total lysates. **c,** IB analysis of indicated GST pull-down precipitates from MCF-7 cells stably expressing HA-CDH1 and treated with DMSO (left) or Sulfopin (right) for 3 days and 10 μM MG132 for last 12 hrs before harvesting. **d,** IB analysis of indicated GST pull-down precipitates from MDA-MB-231 cells stably expressing HA-CDH1 and treated with DMSO or 10 μM Sulfopin for 3 days and 10 μM MG132 for the last 12 hrs before harvesting. **e,** IB analysis for indicated proteins from MCF-7 cells stably co-expressing the APC/C-degron reporter (mCherry-Geminin (1-110)) and

empty vector (EV) or Flag-PIN1 and treated with increasing concentrations of Palbociclib (0.5, 1, 2 μM) for 48 hours. **f,** Percentage of cells in G0/G1 from Fig. 5b. n=3 independent experiments. Data in graphs are mean ± SD and analyzed by unpaired two-sided t-test. p values are shown. **g,** IB analysis of indicated proteins in MCF-7 cells treated with AApin (10 μM ATRA+1 μM ATO) for indicated times. **h, i,** FACS analysis of DNA contents in MCF-7 cells treated as in (**g**) and percentage of cells in G0/G1 and S/G2/M were shown in the curve graph. **j,** Distributions of S/G2/M duration in DMSO-, Palbociclib- and PIN1 inhibitors-treated MCF-7 cells stably expressing the APC-degron reporter from Fig. 5c. Data in graphs are analyzed by unpaired two-sided t-test. p values are shown. **k,** Immunofluorescence for MDA-MB-231 cells stably expressing the APC/C-degron reporter and PIN1 (Venus-PIN1 fusion protein) and treated with different inhibitors for indicated periods. Scale bars, 20 μm. The images were representative images from 3 independent experiments (**a-e, g, k**). Source data are provided as a Source Data file.

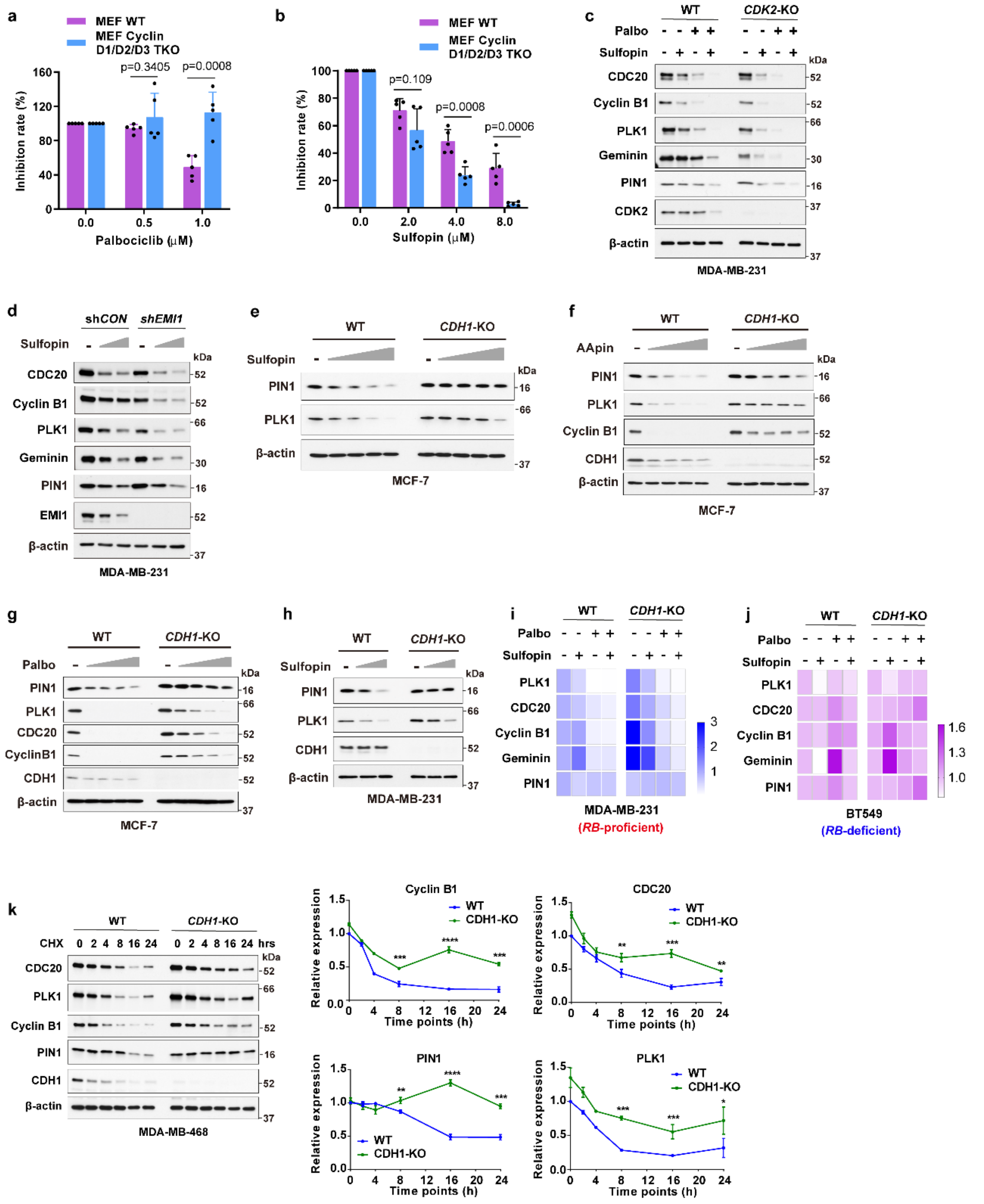

Supplementary Fig. 7 | Pharmacologic inhibition of PIN1 and CDK4 synergistically and irreversibly reactivate APC/C^{CDH1} to induce degradation of PIN1 and other mitotic proteins

a, b, WT and *Cyclin D1/D2/D3* TKO MEFs were treated with Palbociclib (**a**) or Sulfopin (**b**) for 4 days and cell viability were assessed by CellTiter-Glo. n=5 independent experiments. Data in graphs are mean ± SD and analyzed by unpaired two-sided t-test. p values are shown. **c,** IB analysis for indicated proteins from WT and *CDK2*-KO MDA-MB-231 cells treated with 10 µM Sulfopin, 0.5 µM Palbociclib or their combination for 3 days. **d,** IB analysis for indicated proteins from sh*CON* and

sh*EMI1* MDA-MB-231 cells treated with increasing concentrations of Sulfopin (5, 10 μM) for 3 days. **e-g,** IB analysis for indicated proteins from WT and *CDH1*-KO MCF-7 cells treated with increasing concentrations of Sulfopin (2, 4, 8, 10 μM) (**e**), AApin (ATO (0.5, 1, 1.5, 2 μM)+ATRA (5, 10, 15, 20 μM)) (**f**) or Palbociclib (0.5, 1, 2, 4 μM) (**g**) for 3 days. **h,** IB analysis for indicated proteins from MDA-MB-231 cells stably expressing indicated constructs and treated with Sulfopin (5, 10 μM) for 3 days. **i,** RT-PCR analysis of indicated mRNA of WT and *CDH1* KO MDA-MB-231 cells treated with 10 μM Sulfopin, 1 μM Palbociclib or their combination for 3 days. **j,** RT-PCR analysis of indicated mRNA of WT and *CDH1* KO BT-549 cells treated with 5 μM Sulfopin, 2.5 μM Palbociclib or their combination for 3 days. Heatmap represented relative mRNA expression. **k,** (Left) CHX chase assay for indicated proteins from WT and *CDH1*-KO MDA-MB-468 cells treated with 50 μg/ml CHX for the indicated time. Quantification for the relative levels of the indicated proteins. n=3 independent experiments. Data in graphs are mean ±SD and analyzed by unpaired t-test. *$p < 0.05$, **$p < 0.01$, ***$p < 0.001$, ****$p < 0.0001$. The images were representative images from 3 independent experiments (**c-k**). Source data are provided as a Source Data file.

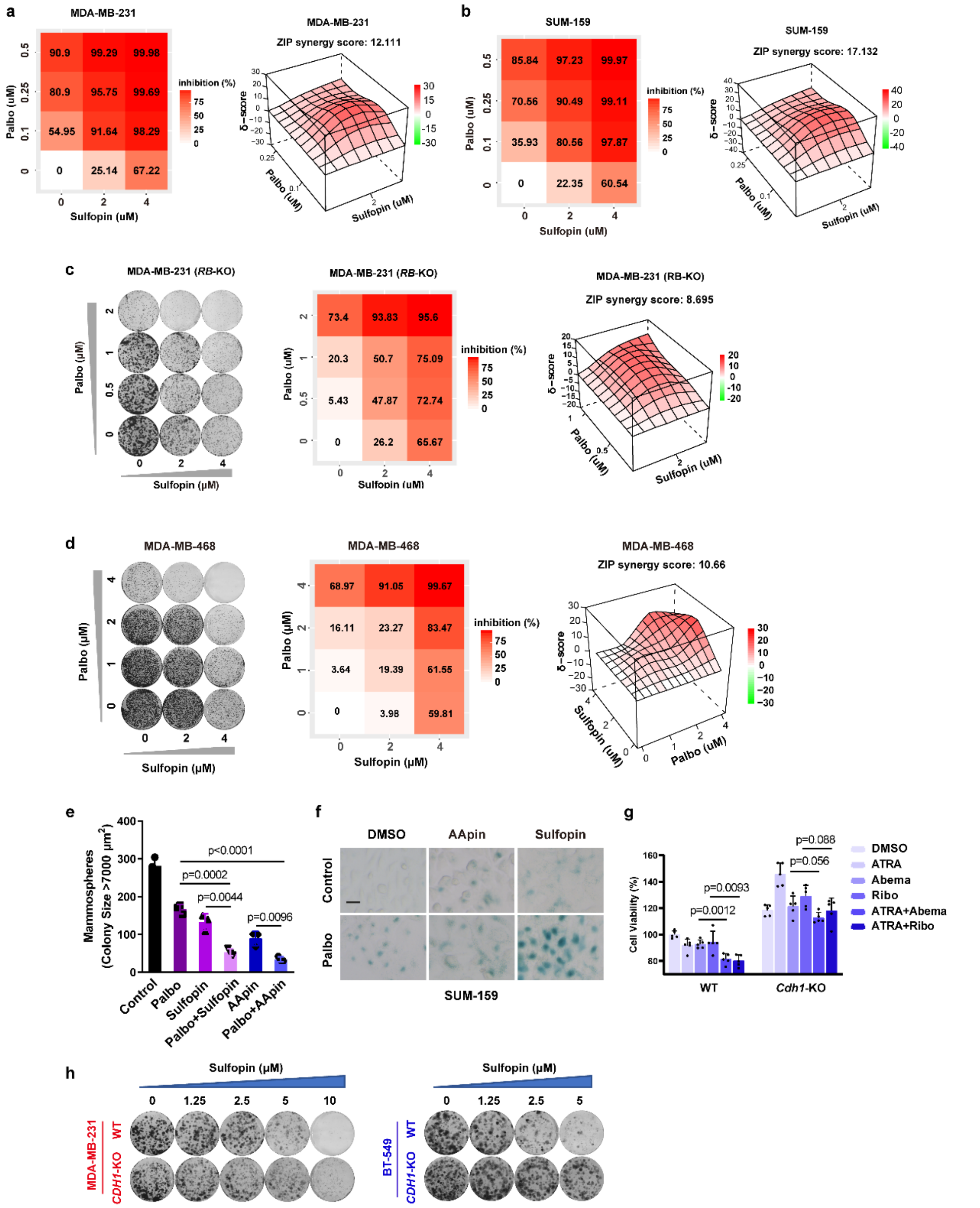

Supplementary Fig. 8 | PIN1 inhibitors synergize with CDK4 inhibitors against TNBC in human cells and immune-compromised mouse models

a, b, The heatmap of growth inhibition matrices and synergy scores for Sulfopin in combination with Palbociclib in MDA-MB-231 cells (**a**) or SUM-159 cells (**b**), higher scores (darker red) denoting stronger synergy. **c, d,** Long-term colony formation assays (left), heatmaps of growth inhibition matrices (middle) and synergy scores (right) for Sulfopin in combination with Palbociclib in *RB*-KO MDA-MB-231 cells (**c**) or MDA-MB-468 cells (**d**). The colonies were stained with crystal violet.. **e,** Mammosphere formation assay of MDA-MB-231 cells treated with 1 µM Palbociclib, 10 µM Sulfopin,

AApin (10 μM ATRA plus 1 μM ATO) or a combination of both drugs for two weeks. n=3 independent experiments. Data in graphs are mean ± SD and analyzed by unpaired two-sided t-test. **f,** Senescence was assessed by staining for SA-β-gal activity for SUM-159 cells treated with AApin (10 μM ATRA+1 μM ATO), 10 μM Sulfopin, 1 μM Palbociclib or combinations for 3 days. Scale bars, 20 μm. **g,** WT and *Cdh1*-KO MEFs were treated with indicated drugs for 3 days and cell viability were assessed by CellTiter-Glo. n=5 independent experiments. Data in graphs are mean ± SD and analyzed by unpaired two-sided t-test. p values are shown (**e, g**). **h,** Long-term colony formation assays of WT and *CDH1*-KO MDA-MB-231 and BT-549 cells treated with indicated Sulfopin doses for two weeks and stained with crystal violet. The images were representative images from 3 independent experiments (**a-d, f, h**). Source data are provided as a Source Data file.

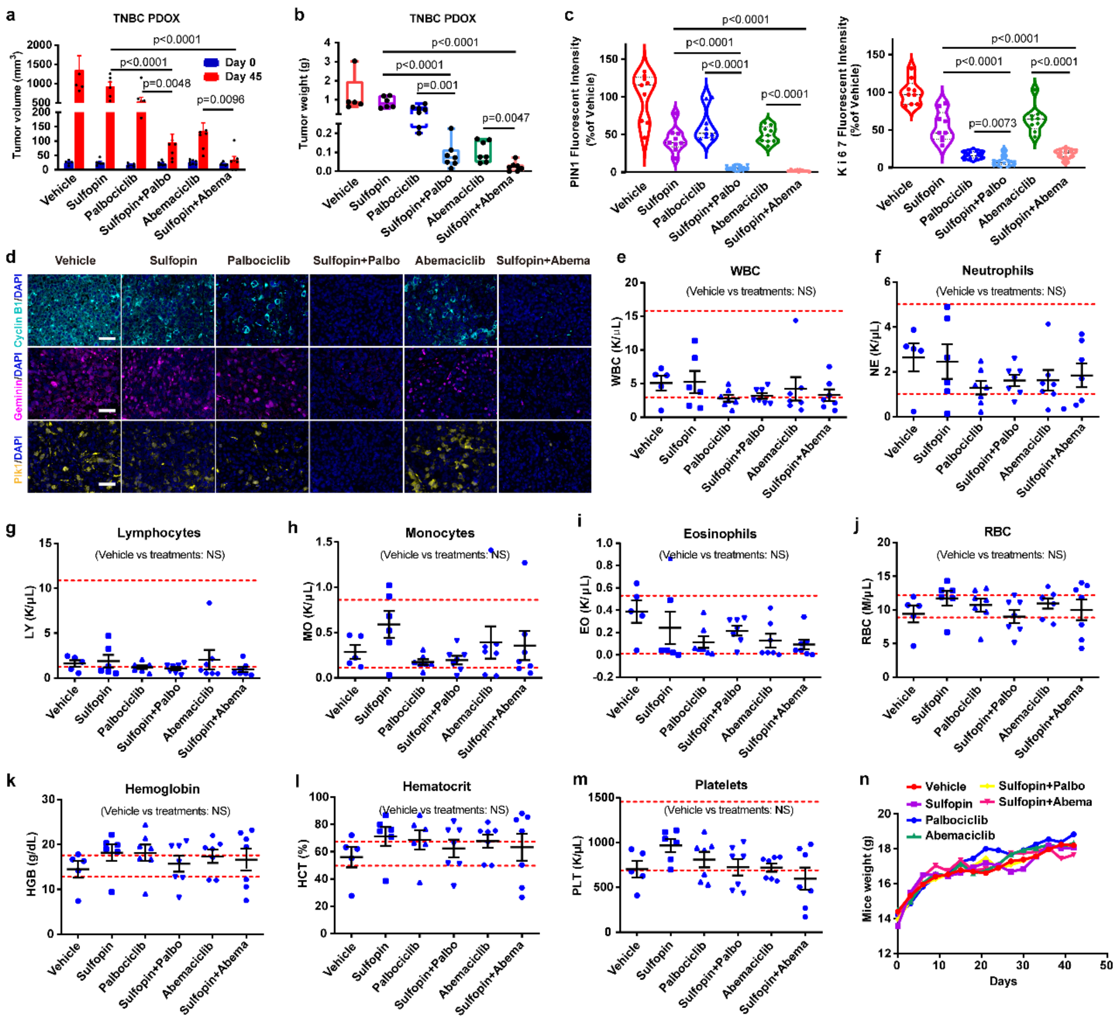

Supplementary Fig. 9 | PIN1 inhibitors synergize with CDK4 inhibitors against TNBC in human cells and immune-compromised mouse models

a, b, Tumor growth in mice with established TNBC PDOX treated with Sulfopin, CDK4 inhibitors or their combination. Tumor sizes (**a**) and tumor weights (**b**) were measured when mice were euthanized after 45 days. Data in graphs are mean ± SEM (**a**). Box plots, the central line marks 50th percentile, the box edges indicate the 25th and 75th percentiles, and the whiskers stretch to the minima and maxima (**b**). **c,** Quantification for the relative levels of PIN1 fluorescent intensity (left) and Ki67 fluorescent intensity (right) from Fig. 6i. Vehicle, n=5 mice; Sulfopin, n=6 mice; Palbociclib, n=7 mice; Abemaciclib, n=7 mice; Sulfopin+ Palbociclib, n=7 mice; Sulfopin+ Abemaciclib, n=7 mice. Data in graphs are analyzed by unpaired two-sided t-test (**a-c**). p values are shown. **d,** Representative immunofluorescence images for PDOX tumors stained with Cyclin B1 (cyan), Geminin (purple) and PLK1 (yellow). Scale bars, 50 µm. **e-m,** Hematological parameters were analyzed in TNBC PDOX nude mice treated with indicated inhibitors for 45 days. Data in graphs are mean ± SEM. Vehicle vs. treatments, not significant (NS). **n,** Body weights were monitored in TNBC PDOX nude mice during the treatments. Source data are provided as a Source Data file.

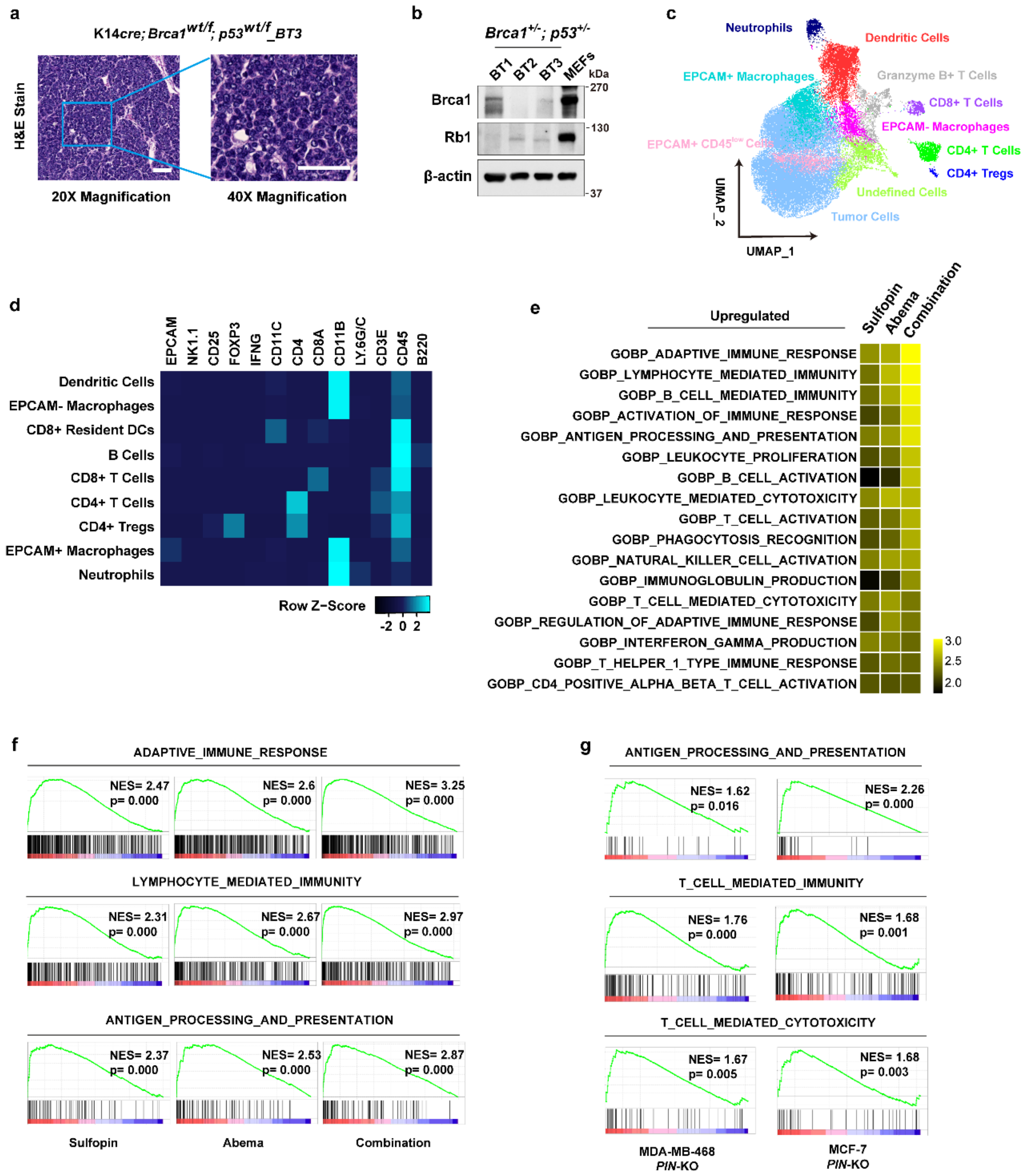

Supplementary Fig. 10 | Combination of PIN1- and CDK4-inhibitors achieves synergistic anti-Tumor immunity and efficacy against *RB*-proficient or -deficient TNBC in immune-competent mouse models

a, Representative images of H&E staining in K14*cre; Brca1wt/f; p53wt/f*. Scale bars, 50 µm. **b,** IB analysis for indicated proteins derived from K14*cre; Brca1wt/f; p53wt/f* tumors or MEFs. The graphs were one representative experiment out of three independent experiments. **c,** Concatenated UMAP plots displaying 40,000 cells derived from K14*cre; p53wt/f; Brca1wt/f* mouse tumors treated with Sulfopin, Abemaciclib or their combination for two weeks and colored by the main cell populations based on manual annotation of PhenoGraph clustering. **d,** Heatmaps showing normalized expression of the indicated markers for PhenoGraph clusters which are grouped by expression profiles. **e,** Normalized counts of each treatment versus vehicle were used

for GSEA analysis against biological process related gene sets. Normalized enrichment scores (NES) were used to generate heatmaps for visualization of the functional transcriptional outputs of each treatment. **f,** Enrichment plots of up-regulated gene sets analyzed by GSEA from (**e**). **g,** Enrichment plots of indicated up-regulated gene sets analyzed by GSEA in *PIN1*-KO versus WT cells. Source data are provided as a Source Data file.

Supplementary Table 1. The relationship between PIN1 protein expression and overall survival using the dataset by Tang et al.

	Cut off: median		Cut off: top quartile	
	HR (95% CI)	*p*	HR (95% CI)	*p*
Model 0	2.67 (1.28-5.57)	0.009	4.56 (1.90-10.95)	<0.001
Model 1: adjusting for age	2.47 (1.18-5.17)	0.016	4.74 (1.97-11.45)	<0.001
Model 2: adjusting for age & ER status	2.21 (1.04-4.70)	0.040	3.92 (1.55-9.90)	0.004
Model 3: adjusting for age, ER status & tumor grade	2.53 (1.09-5.85)	0.031	3.14 (1.15-8.59)	0.026
Model 4: adjusting for age, ER status & PAM50	2.12 (0.91-4.94)	0.082	3.34 (1.17-9.54)	0.025
Model 5: adjusting for age, ER status & PAM50 proliferation score	1.76 (0.75-4.11)	0.192	3.67 (1.31-10.29)	0.014

HR, Hazard ratio; CI, confidence interval; ER. estrogen receptor; PAM50, a 50-gene signature that classifies breast cancer into five molecular intrinsic subtypes; PAM50 proliferation score was computed using 11 proliferation-related genes part of PAM50. Two-sided Wald tests were used for the analysis of individual variable coefficients.

Supplementary Table 2. The characteristics of the human BC specimens

patient ID	ER status	PR status	Duration of response (months)	Best response during treatment	Bx site Pre	Bx site Post
1	pos	pos	6	PR	Breast	Bone
2	pos	weak	3	PD	Breast	Liver
3	pos	neg	19	SD	Lymph Node	left axilary soft tissue mass
4	pos	neg	8	SD	Skin	Bladder
5	pos	pos	12	SD	Liver	Breast
6	pos	pos	23	SD	Breast	Liver
7	pos	neg	11	PR	Lymph Node	Small Bowel
8	pos	weak	1	PD	Breast	Skin
9	pos	neg	9	PD	Brain	Liver

ER – estrogen receptor; PR – progesterone receptor; PD – progressive disease, PR- partial remission; SD – stable disease;

Supplementary Table 3. Genotyping primers and probes for K14*cre; Brca1wt/f; p53wt/f* transgenic mice

BRCA Flox F	GGGATCCACTAGTTCTAGTTAGAGCGG
BRCA Flox R	CCAGGGCTCTTAATCCAATCATTCTCCA
BRCA Flox probe	CAGAGAGCCTGTCTCAA
BRCA WT F	TGGGCTTGCTGACTAGTTATTACAG
BRCA WT R	GCTCTTAATCCAATCATTCTCCACCTTATTT
BRCA WT probe	CAAGCCCCGGGTGCAG
P53 Flox F	CGTATAATGTATGCTATACGAAGTTATCTGCAGCCCG
P53 Flox R	GGCACCTTTGATCCCAGCACA
P53 Flox Probe	TCCAACTGTCTCTGCCTC
P53 WT F	CAGCAGTAACCTCCTGGGAATACTTCA
P53 WT R	CTCAGGAAAACAAATTATGATTCGAAC
P53 WT Probe	CAGTCAGTCGCCCTTTC

Supplementary Table 4. The sequences of the primers used for qRT-PCR analyses

	Forward	Reverse
**Human**		
PLK1	CCTGCACCGAAACCGAGTTAT	CCGTCATATTCGACTTTGGTTGC
CDC20	GCTTTGAACCTGAACGGTTTTG	TCTGGCGCATTTTGTGGTTTT
Cyclin B1	AATAAGGCGAAGATCAACATGGC	TTTGTTACCAATGTCCCCAAGAG
Geminin	AAAACGGAGAAAGGCGCTGTA	GGCGGGCAATTTCATTGTCC
PIN1	GCCTCACAGTTCAGCGACT	ACTCAGTGCGGAGGATGATGT
CDH1	AGCCAACTGGAGCGTGAAC	TCTTTGCCGTTGTCTGAGGTG
RNF219	CAGACCGTGCAGAATGTTACA	TCCTGACCGTATGGCTTAGC
UBR5	GGATGATGAAGATGGAGATGATG	CGCAACAGCTCAGAGTCTCTC
RNF149	TTCCAGTAACGATGACCATAGG	CAGCATCAACATCAATTCCCT
WWP2	TTCGAGAGACTCCAGTGGAAC	GTTGTGGGTTCATCATTCACTG
NEDD4	AGAGAGGCAGGATATCCTTGG	GGTGGTAAATGCACGTTGTG
SMURF2	GACACTGGTTATCAGAGGTTGG	TAAGAGGTCTGCCAGGGCTA
FBXO7	TCATCCACAGATTCAGAGCATTC	CAGAACAGTCAGCTGATTGATACA
UBE3A	GCTGCATGTTCTGCTGCTG	ATACGTCAAGTCACATTCCACG
UBE3B	GGATGCCATACGCTTTGTCTA	TAATCCACAGATTGGGAGAACC
KEAP1	TACGACTGCGAACAGCGAC	GCTGAGCGACTGTCGGAAG
RB	TTGGATCACAGCGATACAAACTT	AGCGCACGCCAATAAAGACAT
RBL1	CTGGACGACTTTACTGCCATC	TCCAACCGTGGGAATAATGCT
RBL2	CCACCCCTCAGATCCAGCA	CGTGTAGCTTTCGCTCATGC
**Mouse**		
Cyclin A1	CAGTTTCCCCAATGCTGGTTG	CCTCTGCATACTCCGTTACGTTA
Cyclin B1	CTTGCAGTGAGTGACGTAGAC	CCAGTTGTCGGAGATAAGCATAG
Cyclin D1	GCGTACCCTGACACCAATCTC	ACTTGAAGTAAGATACGGAGGGC
Cyclin E1	CTCCGACCTTTCAGTCCGC	CACAGTCTTGTCAATCTTGGCA
Pin1	TCAGGCCGGGTGTACTACTTC	CCTTGCTCCTGGTGATCTTTT
