# Supplementary material for: Reciprocal antagonism of PIN1-APC/CCDH1 governs mitotic protein stability and cell cycle entry
Source: Nat Commun. 2024 Apr 15;15:3220. doi: 10.1038/s41467-024-47427-w (PMC11018817; doi:10.1038/s41467-024-47427-w)
Supplement: Supplementary file 3 — Description of Additional Supplementary Files [file 41467_2024_47427_MOESM3_ESM.pdf]

Supplementary Data 1.
The relationship between PIN1 protein expression and overall survival in a Breast Cancer Tissue Microarray (TMA)

Supplementary Data 2.
Full list of PIN1-inteacting proteins identified by mass spectrometry

Supplementary Data 3.
CDH1 peptides identified by mass spectrometry

Supplementary Data 4.
16-Panel Markers for CyTOF

Supplementary Movie 1.
MCF7 cells expressing the APC/C-degron reporter were treated with DMSO and followed for 4 days

Supplementary Movie 2.
MCF7 cells expressing the APC/C-degron reporter were treated with Palbociclib and followed for 4 days

Supplementary Movie 3.
MCF7 cells expressing the APC/C-degron reporter were treated with Sulfopin and followed for 4 days

Supplementary Movie 4.
MCF7 cells expressing the APC/C-degron reporter were treated with AApin and followed for 4 days
