# Supplementary material for: Reciprocal antagonism of PIN1-APC/CCDH1 governs mitotic protein stability and cell cycle entry
Source: Nat Commun. 2024 Apr 15;15:3220. doi: 10.1038/s41467-024-47427-w (PMC11018817; doi:10.1038/s41467-024-47427-w)
Supplement: Supplementary file 12 — Reporting Summary [file 41467_2024_47427_MOESM12_ESM.pdf]

nature portfolio

Corresponding author(s): Kun Ping Lu

Last updated by author(s): March 11, 2024

Reporting Summary

Nature Portfolio wishes to improve the reproducibility of the work that we publish. This form provides structure for consistency and transparency in reporting. For further information on Nature Portfolio policies, see our Editorial Policies and the Editorial Policy Checklist.

Statistics

For all statistical analyses, confirm that the following items are present in the figure legend, table legend, main text, or Methods section.

n/a	Confirmed	
☐	☒	The exact sample size (*n*) for each experimental group/condition, given as a discrete number and unit of measurement
☐	☒	A statement on whether measurements were taken from distinct samples or whether the same sample was measured repeatedly
☐	☒	The statistical test(s) used AND whether they are one- or two-sided<br>*Only common tests should be described solely by name; describe more complex techniques in the Methods section.*
☒	☐	A description of all covariates tested
☐	☒	A description of any assumptions or corrections, such as tests of normality and adjustment for multiple comparisons
☐	☒	A full description of the statistical parameters including central tendency (e.g. means) or other basic estimates (e.g. regression coefficient) AND variation (e.g. standard deviation) or associated estimates of uncertainty (e.g. confidence intervals)
☐	☒	For null hypothesis testing, the test statistic (e.g. *F*, *t*, *r*) with confidence intervals, effect sizes, degrees of freedom and *P* value noted<br>*Give P values as exact values whenever suitable.*
☒	☐	For Bayesian analysis, information on the choice of priors and Markov chain Monte Carlo settings
☒	☐	For hierarchical and complex designs, identification of the appropriate level for tests and full reporting of outcomes
☒	☐	Estimates of effect sizes (e.g. Cohen's *d*, Pearson's *r*), indicating how they were calculated

Our web collection on statistics for biologists contains articles on many of the points above.

Software and code

Policy information about availability of computer code

Data collection	Orbitrap Eclipse Tribrid mass spectrometer (Thermo Scientific) was used to collect proteomics data;<br>Live cell images were acquired using the MetaMorph Software in mageXpress Micro Confocal High-Content Imaging System;<br>Tissue microarray images were captured using the CyteFinder high-throughput imaging system (RareCyte WA);<br>Fluorescent images were captured using BZ-X800 analyzer (KEYENCE) and LAS X software (Leica);<br>CyTOF data were acquired using CyTOF software (Fluidigm Helios);<br>NMR data were acquired on Bruker NEO 600 MHz spectrometer;<br>qRT-PCR data were collected using ROCHE LightCycler 96 Real Time PCR instrument;<br>Flow cytometry data were acquired using CytoFLEX LX1 Flow Cytometer;<br>Cell colonies were counted using Celigo Image Cytometer;<br>Fluorescence intensity for PIN1 activity was measured using BIOTEK Synergy H1.
Data analysis	DIA-MS data analysis was performed using Spectronaut v16;<br>The analysis packages used in the MATLAB R2019b are for automatic segmentation, and quantification of fluorescent reporter cells following live imaging (https://github.com/scappell/Cell_tracking), and p53 Cinema Single Cell Tracking (https://github.com/balvahal/p53CinemaManual);<br>GSEA software 4.2.3 (Broad) was used for Gene set enrichment analysis in RNA-seq;<br>NMRFAM Sparky software (1.414) was used for NMR data analysis;<br>HADDOCK2.2 and PyMOL 4.60 were used for docking model and visualization respectively;<br>FlowJo v10.6.2 was used for CyTOF and Flow cytometry data anaylsis;<br>SynergyFinder was used for calculating synergy score;

Colocalization rates were calculated using the LAS X software (Leica);
Image J was used to quantify the relative intensity of western blots and fluorescent images;
The Database for Annotation, Visualization and Integrated Discovery (DAVID) was used to Identify enriched biological terms;
Data analysis, statistical testing and visualization were conducted in GraphPad Prism 9 or RStudio 4.0.2

For manuscripts utilizing custom algorithms or software that are central to the research but not yet described in published literature, software must be made available to editors and reviewers. We strongly encourage code deposition in a community repository (e.g. GitHub). See the Nature Portfolio guidelines for submitting code & software for further information.

Data

Policy information about availability of data

All manuscripts must include a data availability statement. This statement should provide the following information, where applicable:

- Accession codes, unique identifiers, or web links for publicly available datasets
- A description of any restrictions on data availability
- For clinical datasets or third party data, please ensure that the statement adheres to our policy

Full list of PIN1-inteacting proteins identified by mass spectrometry in this study are provided in Supplementary Data 2. The mass spectrometry raw data generated in this study have been deposited in the ProteomeXchange Consortium under accession code PXD046325 [https://www.ebi.ac.uk/pride/archive/projects/PXD046325]. The PIN1 human breast cancer protein data were obtained from Tang, W. et al. research paper (Genome Med). The data for the correlation of Pin1 and Geminin protein levels were obtained from cBioPortal for Breast Invasive Carcinoma (TCGA, PanCancer Atlas, mass spectrometry by CPTAC). The Cdh1 (FZR1) human breast cancer mRNA data were obtained from UCSC Xena (https://xenabrowser.net/) for GDC TCGA Breast Cancer. The proteomics data of PIN1 KO versus WT MDA-MB-231 cells were obtained from Kozono, S. et al. research paper (Nat Commun). Pin1 (PDB: 1PIN) and Cdh1 (PDB: 4UI9) PDB data are used for docking models. The RNA sequencing data generated in this study have been deposited in the NCBI Gene Expression Omnbus (GEO) under accession codes GSE232285 [https://www.ncbi.nlm.nih.gov/geo/query/acc.cgi?acc=GSE232285] and GSE232422 [https://www.ncbi.nlm.nih.gov/geo/query/acc.cgi?acc=GSE232422]. The remaining data are available within the Article, Supplementary Information or Source Data file.

Research involving human participants, their data, or biological material

Policy information about studies with human participants or human data. See also policy information about sex, gender (identity/presentation), and sexual orientation and race, ethnicity and racism.

Reporting on sex and gender	All specimens were from females as approximately 99% of breast cancer occurs in females. This aligns with the disease's epidemiological profile and concentrate our analysis on the population most impacted by breast cancer. Disaggregated sex and gender information has not been collected.
Reporting on race, ethnicity, or other socially relevant groupings	There is no socially constructed or socially relevant categorization variables used in the manuscript.
Population characteristics	Tissues were from nine female patients diagnosed with metastatic, ER+ BC between 1999 and 2021. Specimens were de-identified and logged in a Red Cap database (Protocol DF/HCC legacy 17-503) and retrieved from the pathology archive. See also Supplementary Table 2.
Recruitment	This protocol uses archival materials. There was no active recruitment of participants. We analyzed available tumors that had corresponding pre- and post-CDK4/6 inhibitor biopsies.
Ethics oversight	The specimen were collected according to protocol DF/HCC 17-503. The protocol is active and approved by the Institutional Review Board (IRB) at Dana Farber Harvard Cancer Center. Given the minimal risk of the study, the requirement for informed consent was waived by the IRB.

Note that full information on the approval of the study protocol must also be provided in the manuscript.

Field-specific reporting

Please select the one below that is the best fit for your research. If you are not sure, read the appropriate sections before making your selection.

☒ Life sciences ☐ Behavioural & social sciences ☐ Ecological, evolutionary & environmental sciences

For a reference copy of the document with all sections, see nature.com/documents/nr-reporting-summary-flat.pdf

Life sciences study design

All studies must disclose on these points even when the disclosure is negative.

Sample size	No formal sample-size calculation was conducted for our study. Instead, the sample sizes were determined based on benchmarks established by previous related studies, specifically those by Kozono, S. et al. (Nat Commun, 2018) and Dubiella, C. et al. (Nat Chem Biol, 2021), which provided a foundation for estimating the number of samples necessary to observe significant biological effects.<br>For the immune-compromised mouse model experiments, 5-7 mice per arm for PDOX models; 5 mice per arm for MDA-MB-468 xenografts; 6 mice per arm for K14cre; p53wt/f; Brca1wt/f_BT3 cohort; 5 mice per arm for K14cre; p53wt/f; Brca1wt/f_BT1 cohort;<br>For the syngeneic mouse model experiments, 10 mice per arm for K14cre; p53wt/f; Brca1wt/f cohorts.<br>These sample sizes were deemed sufficient to yield detectable biological outcomes, with statistical significance being established through

	biologically independent repeats.
Data exclusions	No data were excluded from the analyses.
Replication	We have specified the number of independent biological repetitions conducted for each experiment in the figure legends. For animal studies, a minimum of n=5 mice per group were used to ensure statistical robustness. All replication attempts yielded consistent and similar results.
Randomization	For all in vivo experiments, after tumor reached 3-5mm, mice were randomly assigned into different treatment arms. The starting tumor sizes in the treatment and control arms were similar before treatment. For our in vitro experiments, randomization was not employed as a method of group allocation, due to the nature of these experiments where controlled conditions and specific variables dictate the experimental setup.
Blinding	Immunofluorescence staining and single cell tracking analysis were performed by independent researchers in a blinded fashion. Due to feasibility, investigators were not binded during other experiments.

Reporting for specific materials, systems and methods

We require information from authors about some types of materials, experimental systems and methods used in many studies. Here, indicate whether each material, system or method listed is relevant to your study. If you are not sure if a list item applies to your research, read the appropriate section before selecting a response.

Materials & experimental systems

n/a	Involved in the study
☐	☒ Antibodies
☐	☒ Eukaryotic cell lines
☒	☐ Palaeontology and archaeology
☐	☒ Animals and other organisms
☒	☐ Clinical data
☒	☐ Dual use research of concern
☒	☐ Plants

Methods

n/a	Involved in the study
☒	☐ ChIP-seq
☐	☒ Flow cytometry
☒	☐ MRI-based neuroimaging

Antibodies

Antibodies used	Antibodies for western blot:<br>Anti-Pin1 mouse monoclonal antibody was provided by Dr. Xiao Zhen Zhou (homemade). Anti-Cdh1 (sc-56312, 1:000), anti-Cyclin E (C-19, sc-198, 1:1000) and anti-CDC20 (sc-13162, 1:1000) antibodies were purchased from Santa Cruz. Anti-RB (ab181616, 1:2000), anti-APC7 (ab4171, 1:500) and anti-Thiophosphate ester (ab92570, 1:5000) antibodies were purchased from Abcam. Monoclonal anti-Flag M2 antibody (F1804, 1:10000) from Sigma. Anti-HA-Tag rabbit mAb (3724, 1:1000), anti-HA-Tag mouse mAb (2367, 1:1000), anti-CDK2 rabbit mAb (2546, 1:1000), anti-CDK4 (D9G3E) rabbit mAb (12790, 1:1000), anti-APC8 rabbit mAb (15100, 1:1000), anti-Cyclin B1 antibody (4138, 1:1000), anti-Cyclin A2 mouse mAb (4656, 1:1000), anti-Cyclin D1 rabbit mAb (55506, 1:1000), anti-PLK1 rabbit mAb (4513, 1:1000), anti-Phospho-MAPK/CDK Substrates (PXS*P or S*PXR/K) (2325, 1:1000) and anti-Geminin rabbit mAb (52508, 1:1000) antibodies were purchased from Cell Signaling Technology. Anti-Emi1 mouse mAb (376600, 1:500) was purchased from Thermo.<br>Antibodies for immunoprecipitation:<br>Anti-PIN1 rabbit monoclonal antibody (ab192036, 1:50) and anti-CDK4 antibody (ab68266, 1:50) were purchased from Abcam.<br>Antibodies for Immunofluorescent staining:<br>Anti-PIN1 rabbit monoclonal antibody (ab192036, 1:50) and anti-PanCK (ab86734, 1:100) were purchased from Abcam, Anti-Ki67 (#50-828-02, 1:400) antibody was purchased from Biocare Medical.<br>Antibodies for CyTOF:<br>Metal-conjugated antibodies used for CyTOF were purchased from Fluidigm, the antibodies details (species, catalog number, clone name and usage) were provided in Supplementary Data 4.
Validation	Anti-Pin1 mouse monoclonal antibody was provided by Dr. Xiao Zhen Zhou (homemade) and has been validated in Kozono et al. Nat Commun. 2018, Koikawa et al. Cell. 2021, and this paper.<br>All other antibodies were purchased from reputable manufactures and have been validated for the indicated applications by the manufacturers.

Eukaryotic cell lines

Policy information about cell lines and Sex and Gender in Research

Cell line source(s)	MDA-MB-468, BT-549, MDA-MB-231, MCF-7 and HEK293T cells were obtained from ATCC. Wild-type and Cdh1-/- Mouse embryonic fibroblasts (MEFs) were kind gifts from Dr. Wenyi Wei (BIDMC). MCF-10A cells were gifts from the S. D. Cappell (NIH/NCI). SUM-159 cells were purchased from BioIVT. K14cre;Brca1wt/f;p53wt/f mouse cells were isolated from genetically engineered mouse model (K14cre;Brca1wt/f;p53wt/f).
Authentication	All cell lines, except K14cre;Brca1wt/f;p53wt/f mouse cells, used in this study were authenticated by STR profile report.

Mycoplasma contamination	All cell lines in our laboratory were routinely tested for mycoplasma contamination and cells used in this study were mycoplasma free.
Commonly misidentified lines (See ICLAC register)	No commonly misidentified cell lines were used in this study.

Animals and other research organisms

Policy information about studies involving animals; ARRIVE guidelines recommended for reporting animal research, and Sex and Gender in Research

Laboratory animals	6-week-old female BALB/c nude mice, 6-week-old female FVB/NJ mice,<br>K14cre; Brca1wt/f; p53wt/f female mice with FVB/129P2 mixed genetic background (ages when breast tumors developed)
Wild animals	The study did not involve wild animals.
Reporting on sex	All mice used in this study are female as approximately 99% of breast cancer occurs in females. This aligns with the disease's epidemiological profile and concentrate our analysis on the population most impacted by breast cancer. Disaggregated sex information has not been collected.
Field-collected samples	The study did not involve samples collected from field.
Ethics oversight	Animal studies were approved by Beth Israel Deaconess Medical Center Institutional Animal Care and Use Committee (IACUC; protocol number 052-2020), and performed in accordance with guidelines established by NIH Guide for the care and use of laboratory animals.

Note that full information on the approval of the study protocol must also be provided in the manuscript.

Plants

Seed stocks	Not applicable
Novel plant genotypes	Not applicable
Authentication	Not applicable

Flow Cytometry

Plots

Confirm that:

- [x] The axis labels state the marker and fluorochrome used (e.g. CD4-FITC).
- [x] The axis scales are clearly visible. Include numbers along axes only for bottom left plot of group (a 'group' is an analysis of identical markers).
- [x] All plots are contour plots with outliers or pseudocolor plots.
- [x] A numerical value for number of cells or percentage (with statistics) is provided.

Methodology

Sample preparation	Synchronized cells were collected at the indicated time points and fixed by 75% ethanol at -20°C overnight. After fixation, the ethanol was completely removed via centrifugation, and the cells were washed three times with cold PBS. Then, the cells were resuspended in propidium iodide (PI) staining solution provided by cell cycle kit (Beckman Coulter, C03551) according to the manufacturer's instructions.
Instrument	CytoFLEX LX1 Flow Cytometer
Software	Data acquisition was performed using CytExpert and data analysis was performed using FSC Express software and FlowJo software
Cell population abundance	Not applicable

Gating strategy	No specialized FACS gating strategies were employed for the analysis presented in our manuscript.

☐ Tick this box to confirm that a figure exemplifying the gating strategy is provided in the Supplementary Information.
